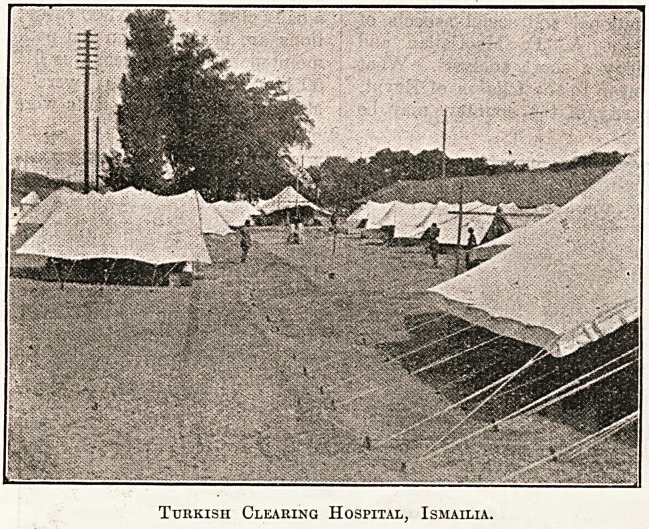# The Vanquishing of Ophthalmia: A Crusade against One of the Plagues of Egypt

**Published:** 1916-01-08

**Authors:** 


### THE VANQUISHING OF OPHTHALMIA.

### A Crusade against One of the Plagues of Egypt.

The third annual report on the work of the Ophthalmic Section of the Public Health Department of the Ministry of the Interior of Egypt has recently been published, and is a document worthy of careful study by economists, philanthropists, and statesmen, as well as by those interested in ophthalmology and in tropical hygiene. It deals with the year 1914, and contains much valuable information about the bacteriology of Egyptian ophthalmia, much of which is to all intents research work. Into this it is not necessary here to enter; it is rather the institutional and social aspects of the work done by Dr. A. F. MacCallan and his staff that will repay a short analysis. What ophthalmic diseases mean to the *fellahin* of Egypt, and so to the prosperity of the country, may be gauged by the fact that 14 per cent. of those who were examined at the ophthalmic hospitals were blind in one or both eyes. This percentage is terribly high, but it shows a steady improvement since the ophthalmic department got to work, for in 1913 it was 15, and in 1912, 16. Five per cent. of those under treatment were infants under one year, and 38 per cent. were children under fifteen. Hundreds of children are being saved every year from blindness; but, as the Director remarks, "the importance of early treatment has yet to be realised by the majority of Egyptian mothers."

Travelling Ophthalmic Hospital, Delta Barrage.

### Hospital Organisation.

The outbreak of war has seriously interfered with the expansion and progress of the anti-ophthalmic campaign. Besides the recall to active service of two of the British surgeons, the Department has suffered disorganisation also through the demands for clearing and base hospitals. Two of the former and one of the latter have been arranged and are being administered by the Director and his staff; eight hundred beds for the military have thus been provided, and this has involved the conversion of the travelling hospitals into permanent institutions for general surgery, not for eye work. The full effect of this conversion will no doubt be reflected in next year's report; the present one refers to the period before this dislocation of the arrangements.

By the end of 1914 sixteen ophthalmic hospitals had come into existence in Egypt. Two were endowed by Sir Ernest Cassel with a capital sum of £40,000; four are maintained by local self-taxation, eight by Government, and two are closed for lack of funds. It is found by experience that a satisfactory hospital can be built for about £4,000, comprising a commodious out-patient department and sixteen beds for in-patients. This suffices for a daily clinic of 200 to 300 cases; most of the operations are performed on out-patients. The cost of maintenance for the year was £12,400; for this sum 50,126 new patients were treated; 686,012 attendances by out-patients were registered; 2,071 in-patients were treated; and 40,710 operations were performed. The staff (before the war) consists of five European and twenty-seven native Egyptian surgeons; the enormous experience which they gain renders the senior surgeons among the latter very expert operators. The two Cassel Fund hospitals are travelling hospitals, which pitch their tents for about six months in a locality and then move on elsewhere to meet urgent needs.

### Statistical and Clinical.

No fewer than 11,955 eyes were found to be blind during the year 1914. Just over 9,000 of these were due to various kinds of conjunctivitis, including trachoma, while glaucoma accounted for 1,150 more, and cataract for 862. The total number of cases of senile cataract encountered was 1,159; but only 449 were operated upon, because a large proportion were complicated by glaucoma, trachoma, corneal opacity, or other condition rendering extraction of the cataract useless. Glaucoma, as will be gathered from the fact that 1,761 cases of it were encountered, is exceedingly common; the cause of this prevalence is undecided, but the Director takes the view that anatomical peculiarities of the eye in Egyptians may be a predisposing factor. Trephining with iridectomy is the operation chiefly performed for this condition; this operation, introduced in India a few years ago, has superseded the older operation of simple iridectomy almost entirely. The frequency with which the disease arises successively in the two eyes has induced the Director to advise a prophylactic operation in the unaffected eye immediately a diagnosis of glaucoma has been made in the other.

### The Bacteria of Conjunctivitis.

It is, however, the varied forms of conjunctivitis which are the most important of the eye diseases of Egypt. By dint of careful bacteriological examinations a satisfactory basis for the routine treatment of acute ophthalmias has been secured. It is somewhat surprising to learn that the gonococcus accounts for 43.6 per cent. of them, whereas *Bacillus diphtheriæ* is present in but 0.06 per cent. The Morax-Axenfeld and Koch-Weeks bacilli stand next in point of frequency to the gonococcus. Chronic and subacute gonococcal conjunctivitis occurs during the winter; acute forms of this disease mainly in the hot weather; and various kinds of mixed infections are very frequently found.

Turkish Clearing Hospital, Ismailia.

### Trachoma.

In the same way a classification of trachoma, and a routine line of treatment based thereon, have been adopted. The ravages of this disease may be estimated from the fact that over 14,000 operations for trichiasis-entropion, its sequela, were performed. It is in the schools and among the children generally that this veritable scourge can be attacked with the best prospect of cure; and accordingly much attention is paid to a system of inspection and treatment of school children. This has been introduced in eight primary schools in important towns, and it is hoped ultimately to extend it very greatly. The proportion of pupils affected by trachoma is now 92 per cent. In 1907 the schools contained 95.5 per cent. of pupils in the more infective stages of this complaint; but that is now reduced to 11.7 per cent., and there seems reason to hope that when financial difficulties can be overcome this very serious detriment to the health, happiness, and prosperity of the Egyptians will be satisfactorily dealt with and perhaps even eradicated.

The first stage of trachoma is described as that of early infection, characterised by slight roughness of the inner surface of the eyelid with greyish dots. In this stage silver nitrate (2 per cent.) and copper sulphate stick are used. The second stage is sub-divided into three: one characterised by greyish follicles which can be ruptured by pressure, another in which raspberry-like papillæ mask the typical follicles; and a third in which gonococcal conjunctivitis has supervened as a complication. Rupture of the follicles, scraping, and perchloride of mercury (1 per cent.) are advised for the first two of these types, with Heisrath's combined excision of the tarsus and conjunctiva in certain cases; silver nitrate (2 per cent.) is the usual treatment for the third type. The third stage is that of commencing cicatrisation, to be treated by copper sulphate stick and by incision with a knife of any spots of degeneration. In the fourth and last stage of complete cicatrisation treatment of the trachoma itself is no longer possible, though that of the resulting deformity of entropion is, as has been indicated, very often required. Snellen's operation was done 11,474 times, and Van Millingen's 2,701 times.

An ophthalmic inspection of the children in the infant schools of Tanta and Assiut has been carried out annually for some years. Thirty-nine such schools were inspected during the year, and 3,129 pupils were examined in detail. About one-third of the schools were "definitely dirty"; the report does not say whether any were "definitely clean." Over 94 per cent. of the children were affected with trachoma: of the pupils whose vision could be accurately taken, one-half at Tanta and one third at Assiut had bad vision. The blind in one eye were 2.3 and 4.4 per cent. respectively in the two districts; 0.7 per cent. were blind in both eyes. No attempt at amelioration of this shocking condition of affairs has been made, though the Director feels he could bring about a great improvement if means for a proper campaign were provided. It is a veritable lesson in statecraft, no less than in clinical medicine, to read through this brief and businesslike report.

## Figures and Tables

**Figure f1:**
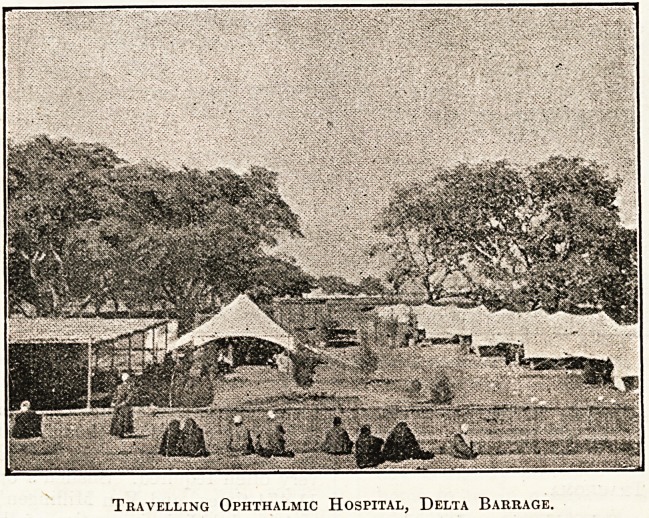


**Figure f2:**